# Tea Polyphenol Protects the Immune Barrier and Inhibits TLR2/NF-κB/MLCK Signal Activation to Prevent Inflammatory Injury in the Intestines of Common Carp (*Cyprinus carpio* L.)

**DOI:** 10.3390/ani15030387

**Published:** 2025-01-30

**Authors:** Man Qian, Jie Yang, Yao Xue, Jiawei Wu, Ziyi Li, Jilong Luo, Bing Zhao, Xuejiao Gao

**Affiliations:** College of Veterinary Medicine, Northeast Agricultural University, Harbin 150030, China; qm18437916979@163.com (M.Q.); yang11jie2022@126.com (J.Y.); 15561850067@163.com (Y.X.); jiayou0609@163.com (J.W.); s230601010@neau.edu.cn (Z.L.);

**Keywords:** tea polyphenol, intestinal immunity, inflammatory injury, TLR2/NF-κB/MLCK pathway

## Abstract

Tea polyphenol (TP) is a kind of natural macromolecular compound present in tea extract with rich biological potential. The purpose of this study was to explore the protective effect of TP on the intestinal immune barrier and the related mechanisms of TP alleviating intestinal injury. Models of common carp (*Cyprinus carpio* L.) and primary intestinal epithelial cells treated with TP and lipopolysaccharide (LPS) were established. The qPCR and ELISA results showed that TP increased the levels of lysozyme (LZ), alkaline phosphatase (ALP), complement component 3 (C3), complement component 4 (C4), immunoglobulin T (IgT), immunoglobulin D (IgD), and immunoglobulin M (IgM) to activate intestinal immune ability. Molecular docking indicated that TP had a strong interaction with TLR2. Meanwhile, TP alleviated LPS-induced intestinal inflammatory damage as evidenced by reducing the mRNA levels of *TNF-α*, *IL-6*, *IL-1β*, *TLR2*, *MyD88*, *P65*, and *IκBα*, which were consistent with those of ELISA and Western blotting results. Moreover, the qPCR and Western blotting results revealed TP promoted the levels of tight junction-related proteins (claudins, occludin, and ZOs) and inhibited the phosphorylation of MLC, which showed the opposite trend after LPS treatment. In summary, the present study indicated that TP improved immune ability and inhibited the activation of the TLR2/NF-κB/MLCK pathway to attenuate LPS-induced inflammatory injury in the intestines of common carp.

## Simple Summary

Tea polyphenol (TP) has increasingly attracted wide, attractive attention due to their powerful biological activities. In this study, we found that TP activated intestinal immune ability and, combined with TLR2 to inhibit NF-κB activation, reduced the expression of pro-inflammatory factors. TP also reserved the phosphorylation of MLC to upregulate the expression of tight junction proteins. These results showed that dietary TP protected the intestinal immune barrier and alleviated inflammatory injury through the TLR2/NF-κB/MLCK signal in the intestines of common carp.

## 1. Introduction

Common carp (*Cyprinus carpio* L.), one of the most consumed in Asia, occupies an important position in freshwater aquaculture. The intestines are an essential immune organ of fish [1]. Intestinal immunity depends on innate and adaptive immune responses. The innate immune components of the intestine include alkaline phosphatase (ALP), lysozyme (LZ), and complements (such as C3 and C4) in fish. LZ exhibited antibacterial effects by destroying bacterial cell walls to lyse bacteria [2]. Complement, equally as an important antimicrobial component, can directly participate in body defense [3]. Igs are the main adaptive immune components of the intestine. Igs (IgM, IgD, and IgT) act as a local immune system to directly combine with the antigen, preventing pathogens from invading the cell in teleosts [4]. Previous studies have shown that LPS injections can increase complement activity to stimulate immune response and induce inflammatory response in carp [5]. In this study, LPS challenge was used to investigate its effect on intestinal immune level.

Toll-like receptors (TLR) are important protein molecules involved in innate immunity, which are also a bridge connecting innate immunity and adaptive immunity. In common carp, TLR2 is a major protein molecule that participates in the inflammatory signal transduction process [6]. The activation of TLR2 is dependent on the combination with the downstream protein myeloid differentiation factor 88 (MyD88) [7]. Nuclear factor kappa-B (NF-κB) is the hub of the downstream signaling pathway of TLR2. The expression of inflammatory factors is regulated by activated NF-κB, which initiates the immune response [8]. However, excessive inflammatory factors are the key triggers of intestinal mechanical barrier destruction. Tight junction proteins are essential components of the mechanical barrier of the intestine. They close the gaps between cells to prevent harmful substances from leaking into the body [9]. It is now understood that tight junction proteins are regulated by myosin light chain kinase (MLCK). MLCK promotes myosin light chain (MLC) phosphorylation and affects the expression of tight junction proteins, resulting in intestinal barrier dysfunction [10]. In addition, the activation of MLCK is regulated by NF-κB, which affects tight junctions, causing increased intestinal permeability [11].

Tea polyphenol (TP), the main active ingredient in tea, is widely used as food additives [12]. TP has been proven to have a variety of biological activities, including anti-inflammatory, antioxidant, and immune protection effects [13,14]. Studies have pointed out that dietary TP can improve the growth performance of groupers under a high-fat diet [15]. Studies have shown that TP can also alleviate tissue damage caused by poisons such as tetrabromobisphenol A, trimethyltin chloride, and acetochlor [12,16,17]. Although studies have pointed out the protective effect of TP on fish, the intestinal immune capacity and tissue protection of TP still need to be further explored in common carp. This study attempted to explore the beneficial effect of TP on the intestines by detecting histopathological changes, immune factor levels, inflammation, and tight junction indicators in the intestines of common carp, providing a theoretical basis for the application of TP in aquaculture.

## 2. Materials and Methods

All the experimental procedures were followed by the Institutional Animal Care and Use Committee of Northeast Agriculture University in this study (NEAU2023046).

### 2.1. Molecular Docking

Molecular docking was used to evaluate the interaction between TP and TLR2. AutoDock (v4.2.6) (http://autodock.scripps.edu/) is the automatic docking procedure for this docking study. The GenBank login number of TLR2 is BAU98381. Pymol (v3.1) software was used to optimize the target, such as removing water molecules and small molecular ligands. Hydrogenation and charge treatment were performed with AutoDock Tools and saved in a “pdbqt” format. The binding energy between TP and TLR2 was calculated according to AutoDock’s interaction energy protocol. A binding energy of less than −5.0 kcal/mol represents an ability to interact, and a binding energy of less than −7.0 kcal/mol represents a strong binding activity.

### 2.2. Animals and Treatments

Eighty common carps (mean initial weight: 152.63 ± 11.52 g) were randomly divided into eight tanks (70 × 50 × 30 cm) and fed on a pellet diet at 3% of their body weight two times every day. Continuous 24 h aeration was supplied to the tanks by air-stones. One-third of the water was replaced with clean dechlorinated water and adaptive feeding for 7 days. After 7 days of acclimation, the fish were randomly distributed into four tanks (20 fish/tank). In the control group (CG), the common carp were fed with a control diet for 21 d and intraperitoneal injection with PBS for 96 h. In the TP group (TP), the common carp were fed with a diet containing 1000 mg/kg TP for 21 d and intraperitoneal injection with PBS for 96 h. In the LPS group (LPS), the common carp were fed with a control diet for 21 d and intraperitoneal injection with 4 mg/kg LPS for 96 h. In the TP + LPS group (TL), the common carp were fed with a diet containing 1000 mg/kg TP for 21 d and intraperitoneally injected with 4 mg/kg LPS for 96 h. The concentrations of LPS and TP were determined based on previous studies [16,18]. Other environmental conditions were consistent with natural conditions. Subsequently, the common carp were anesthetized with 0.02% MS-222 and euthanized. The intestines were removed, a part of the intestines rinsed with PBS, and fixed in 4% paraformaldehyde. The remaining intestinal segments were frozen in liquid nitrogen and stored at −80 °C for later tests.

### 2.3. Cell Culture

Primary intestinal epithelial cells of common carp were cultured according to the previous study [19]. Common carp were starved, and intestinal segments were aseptically removed. The intestinal segments were rinsed with PBS containing penicillin (200 μg/mL) and streptomycin (200 μg/mL). Then, intestinal segments were cut into pieces and washed with PBS as described above. The intestinal fragments were digested with trypsin solution for 15 min in a 37 °C water bath. After termination of digestion, the mixture was filtrated through a 100 μm strainer and centrifuged. The cell pellet was resuspended in M199 culture medium containing 10% fetal bovine serum, 100 μg/mL penicillin, and streptomycin and cultured in 6-well plates at 27 °C in a 5% CO_2_ atmosphere.

The cell viability was detected with a Cell Counting Kit-8 (Biosharp, Hefei, China). Intestinal epithelial cells (1 × 10^4^) were seeded in 96-well plates and treated with TP (10, 20, 40, 80, 160, 320 μg/mL) for 48 h. The CG received 0.1% DMSO (*v*/*v*). Furthermore, 10 μL of Cell Counting Kit-8 solution was added to each hole and incubated at 37 °C for 1 h. The absorbance was detected at a wavelength of 450 nm. Relative cell viability was displayed as an absorbance value relative to the control. The viability of primary intestinal epithelial cells could reach more than 90% after being treated with 160 μg/mL TP (Figure S1). Then, 160 μg/mL TP was used for subsequent experiments. LPS (30 μg/mL) concentrations were determined according to previous studies [20].

### 2.4. Histopathology Observation

The intestinal tissue was fixed with 4% glutaraldehyde and then removed, dehydrated in graded ethanol, permeabilized in xylene, embedded in paraffin, and sectioned into 5-micrometer-thick slices. The slices were stained with H&E and observed with a microscope (Olympus, Tokyo, Japan).

### 2.5. Real-Time Quantitative PCR

The total RNA of intestinal tissue and cells were extracted by the Trizol method. A UV spectrophotometer was used to measure the concentration of total RNA. After normalizing the concentration, the RNA was reverse transcribed to cDNA and stored at −20 °C. Primers (Table S1) were designed with Primer 5.0 software, and sequence specificity was verified through BLAST analysis. The expressions of target genes were detected by qPCR, using GAPDH as the housekeeping gene. The reaction mixture had a volume of 20 μL, and the reaction conditions were as follows: denaturation at 94 °C for 30 s, annealing at 55 °C for 30 s (for 40 cycles), and extension at 72 °C for 5 min. The data were treated with a 2^−ΔΔCt^ relative expression method.

### 2.6. ELISA Assay

The expression levels of lysozyme (LZ), alkaline phosphatase (ALP), complement component 3 (C3), complement component 4 (C4), immunoglobulin T (IgT), immunoglobulin D (IgD), and immunoglobulin M (IgM) were detected by ELISA kits (Mmbio, China). ELISA kits purchased from Andy Gene (Beijing, China) were used to detect the levels of interleukin-6 (IL-6), interleukin-1β (IL-1β), and tumor necrosis factor-α (TNF-α). The intestinal tissue was homogenized in precooled PBS at a ratio of 1/9 (*w*/*v*) and centrifuged at 7500 rpm for 15 min. Then, the supernatant was collected, and the remaining operations were carried out by the instructions of ELISA kits. A microplate reader was used to detect the absorbance at a wavelength of 450 nm.

### 2.7. Western Blotting Assay

The intestinal tissue and cells were homogenized in lysis buffer at a ratio of 1/9 (*w*/*v*). The supernatant was collected by centrifugation after remaining on ice for 30 min. The protein concentration was determined by the BCA kit, adjusting to 5 μg/μL. Protein samples were separated by appropriate concentrations of SDS-PAGE (Servicebio, Wuhan, China) at 80 V for 30 min and 120 V for 60 min and transferred to NC membranes at 200 mA. The NC membranes were blocked in 5% skimmed milk for 2 h at room temperature and incubated with the primary antibody (Table S2) at 4 °C overnight. The corresponding secondary antibodies were used to incubate for 2 h at room temperature. Finally, the BCL luminescent solution was used to observe the membranes, and gray analysis was performed.

### 2.8. Protein–Protein Interaction (PPI) Analysis and Radar Plot

To explore the link between intestinal immune molecules and tight junctions, the STRING database was used to establish a PPI network. This database provides possible potential interactions between the encoded proteins. Enter the name of related genes or proteins, and the website will display the interaction of the related proteins [21].

Radar plot is a simple multivariate graphic method. It allows the visual integration of measured biomarkers. The data of multiple indexes are mapped to coordinate axes, which start from the same center point and end at the periphery of the circle. Connecting the points of the same treatment group forms a radar plot [22]. Hiplot tools were used to create a radar plot in this study [23].

### 2.9. Statistical Analysis

All data in this experiment were analyzed with GraphPad Prism 8.0.1 and SPSS 26.0 software. The experiments were repeated at least three times. Shapiro–Wilk was used to test the normal distribution of the data, and Brown–Forsythe was used to perform the homogeneity of variance. The data were performed as the mean ± standard deviation (mean ± SD). A one-way analysis of variance (ANOVA) was performed to analyze the differences between groups. Furthermore, *p*  <  0.05 indicates significant differences.

## 3. Results

### 3.1. TP Relieves LPS-Induced Intestinal Tissue Damage

**Figure 1:**
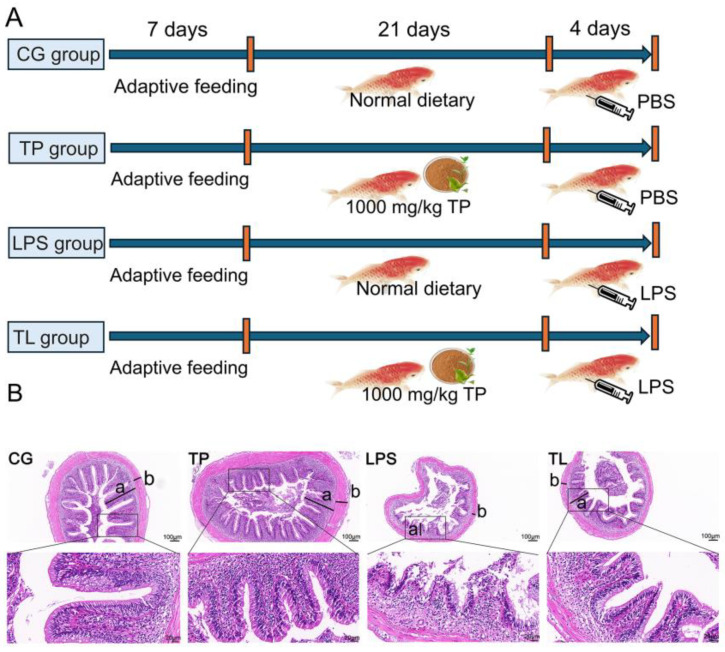
Effects of dietary TP on the intestinal pathological damage challenge by LPS in common carp. (**A**) Schematic diagram of the grouping and treatment on common carp. (**B**) Histopathological staining on the intestines of common carp. a represented the height of the mucosal fold, and b represented the thickness of the muscular layer. The scale bars were 100 μm and 20 μm.

Common carp were given different treatments after seven days of adaptive feeding. Four groups were set up to observe whether TP has positive effects on the intestines under LPS challenge (Figure 1A). Histopathological staining was used to assess tissue damage, and the results are shown in Figure 1B. The intestinal mucosa was intact, and the villi were arranged in order in the CG and TP groups. Severe intestinal injury occurred in the LPS group, including loss and abscission of intestinal villi. Remarkably, the TL group showed a complete histological structure, and the intestinal villi were intact. Moreover, intestinal morphology indexes were also detected. The mucosal fold height and muscular layer thickness of the LPS group were significantly lower, which were increased in the TL group (Figure S2).

### 3.2. TP Enhances the Intestinal Immune Ability

**Figure 2:**
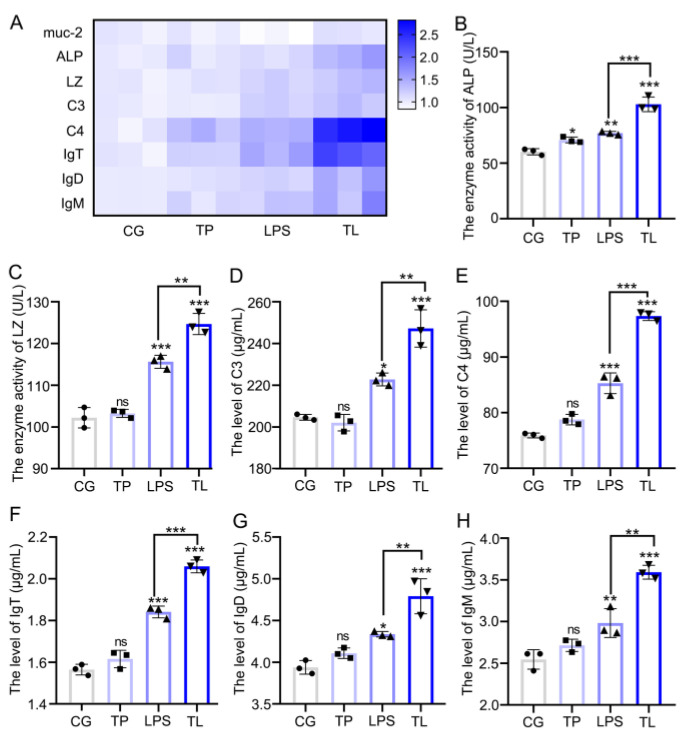
Effects of TP on the intestinal immunity parameters in common carp. (**A**) The mRNA levels of *muc-2*, *ALP*, *LZ*, *C3*, *C4*, *IgT*, *IgD*, and *IgM* in intestinal tissue. (**B**,**C**) The enzymatic activity of ALP and LZ in intestinal tissue. (**D**–**H**) The protein expression levels of C3, C4, IgT, IgD, and IgM in intestinal tissue. * *p* ≤ 0.05, ** *p* ≤ 0.01, *** *p* ≤ 0.001, ns *p* > 0.05.

In response to stimulation, the intestinal immune system could be activated via increasing amounts of immune index, such as LZ, ALP, C3, C4, and Igs [24]. Figure 2 shows the changes in immune index after TP and LPS treatment. The mRNA levels of *mucin-2* (*muc-2*), *ALP*, *LZ*, *C3*, *C4*, *IgT*, *IgD*, and *IgM* were increased in the TL group compared with that in the LPS group (Figure 2A). The enzyme activity of ALP and LZ was significantly increased after LPS stimulation than in the CG. However, the TP group showed higher enzyme activity than the LPS group (Figure 2B,C). LPS stimulated the levels of C3 and C4, with further increasing levels of C3 and C4 in the TL group (Figure 2D,E). Moreover, the levels of IgT, IgM, and IgD have different degrees of increase after LPS treatment compared to the CG; the TL group showed higher levels of Igs compared with the LPS group (Figure 2F–H). These results indicated that TP promoted the immune ability in the intestines of common carp.

### 3.3. TP Relieves LPS-Induced Intestinal Inflammatory Injury

**Figure 3:**
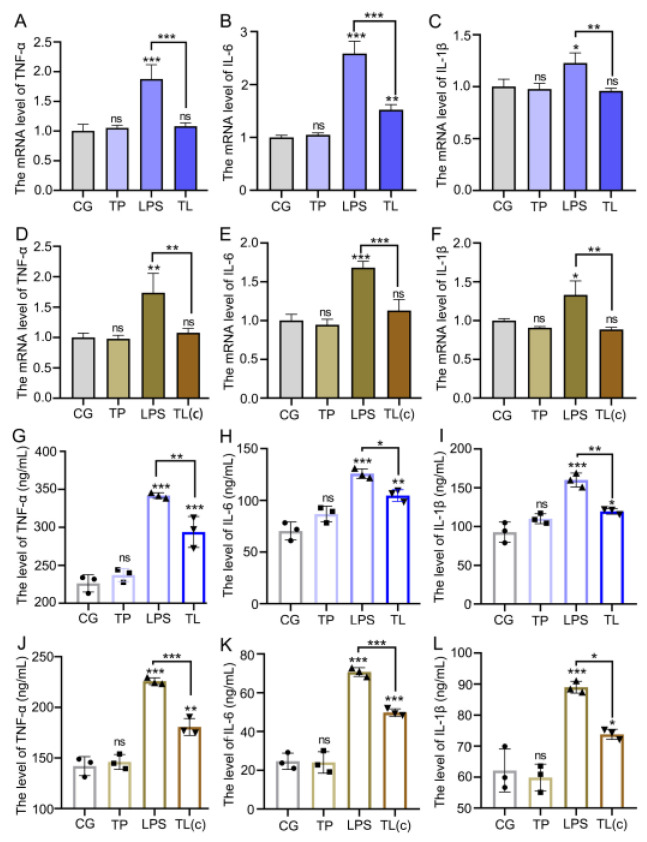
Effects of TP on LPS-induced intestinal inflammatory injury in common carp. (**A**–**C**) The mRNA levels of *IL-6*, *IL-1β*, and *TNF-α* in intestinal tissue. (**D**–**F**) The mRNA levels of *IL-6*, *IL-1β*, and *TNF-α* in intestinal epithelial cells. (**G**–**I**) The protein expression levels of IL-6, IL-1β, and TNF-α in intestinal tissue. (**J**–**L**) The protein expression levels of IL-6, IL-1β, and TNF-α in intestinal epithelial cells. * *p* ≤ 0.05, ** *p* ≤ 0.01, *** *p* ≤ 0.001, ns *p* > 0.05.

The inflammatory cytokines of the intestines and cells were detected by qPCR and ELISA. Compared with the CG, LPS significantly increased the mRNA expression levels of *IL-6*, *IL-1β*, and *TNF-α*. On the contrary, the TL group decreased the mRNA expression levels of these pro-inflammatory factors compared with the LPS group in intestinal tissue and cells (Figure 3A–F). The protein expression level of pro-inflammatory factors was consistent with the mRNA expression levels (Figure 3G–L). These results showed that TP alleviated the intestinal inflammatory injury caused by LPS in common carp.

### 3.4. TP Inhibits LPS-Induced Activation of the TLR2/NF-κB Pathway

**Figure 4:**
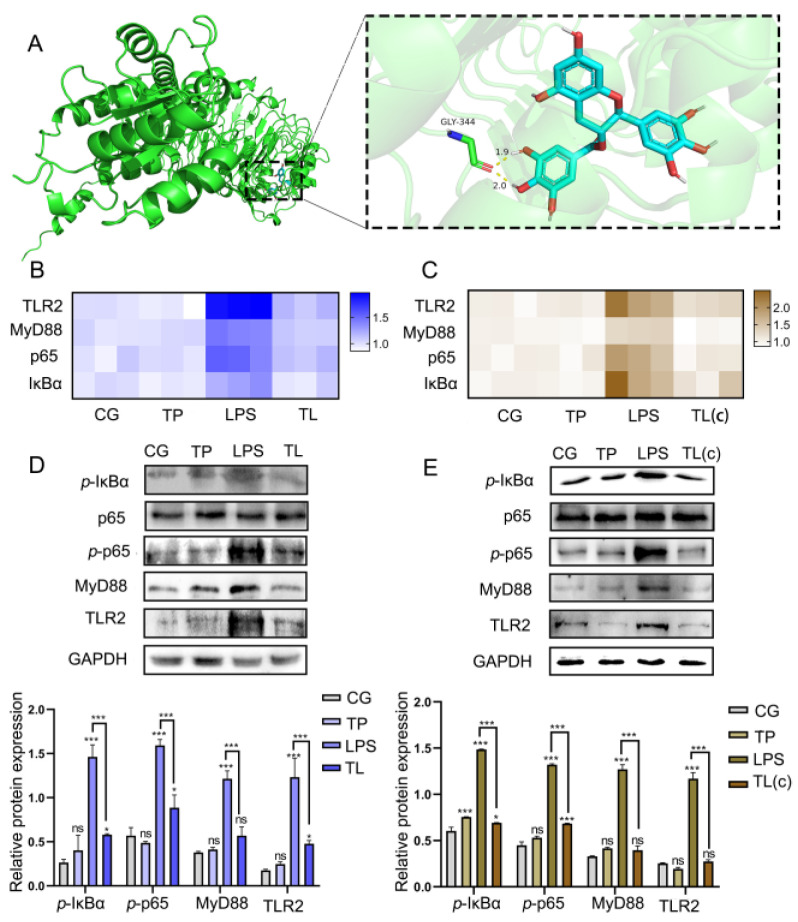
Effects of TP on the TLR2/NF-κB pathway in the intestines of common carp. (**A**) Molecular docking was used to detect the interaction between TP and TLR2. (**B**) The mRNA levels of *TLR2*, *MyD88*, *NF-κB p65*, and *IκBα* in intestinal tissue. (**C**) The mRNA levels of *TLR2*, *MyD88*, *NF-κB p65*, and *IκBα* in intestinal epithelial cells. (**D**,**E**) The protein expression levels of *p*-IκBα, p65, *p*-p65, MyD88, and TLR2 in intestinal tissue and cells. * *p* ≤ 0.05, *** *p* ≤ 0.001, ns *p* > 0.05.

To detect whether TP interacted with TLR2, the AutoDock tool was used to build the model of the TP-TLR2 complex through homology modeling and ligand docking. As shown in Figure 4A, TP mainly interacts with TLR2 through hydrogen bonds. TP formed hydrogen bonds with GLY-334, with hydrogen bond lengths of 1.9 Å and 2.0 Å. The binding energy of TP and TLR2 was −7.9 kcal/mol, which proved that TP had a strong binding activity with TLR2. Subsequently, the expression levels of inflammatory pathway-related factors were detected. The mRNA levels of *TLR2*, *MyD88*, *NF-κB p65*, and *IκBα* increase remarkably after being treated with LPS compared with the CG in tissue and cells. However, these indicators significantly decreased in the TL group (Figure 4B,C). The protein expression levels of TLR2, MyD88, *p*-p65, and *p*-IκBα showed a similar trend with mRNA levels (Figure 4D,E, Figure S3). These results indicated that TP significantly inhibited the activation of the TLR2/NF-κB pathway.

### 3.5. TP Relieves LPS-Caused Destruction of Tight Junction

**Figure 5:**
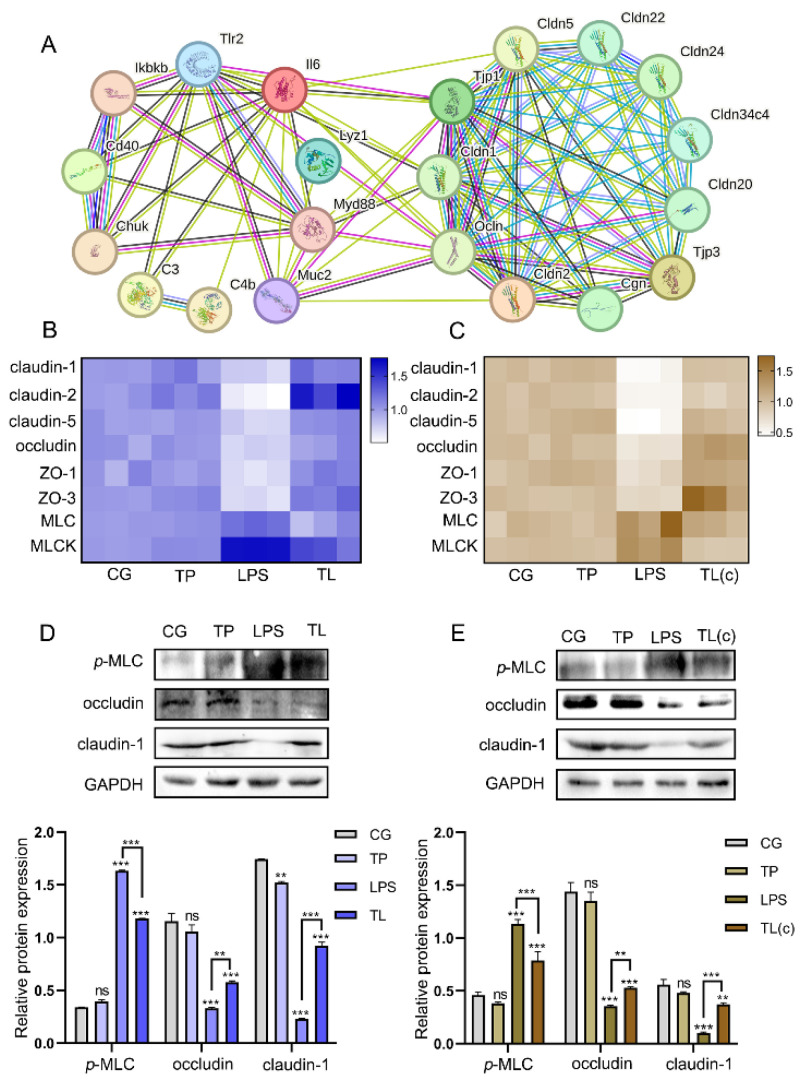
Effects of TP on the intestinal tight junction parameters in common carp. (**A**) The PPI network between intestinal immune factors and tight junctions. (**B**) The mRNA levels of *claudin-1*, *claudin-2*, *claudin-5*, *occludin*, *ZO-1*, *ZO-3*, *MLC*, and *MLCK* in intestinal tissue. (**C**) The mRNA levels of *claudin-1*, *claudin-2*, *claudin-5*, *occludin*, *ZO-1*, *ZO-3*, *MLC,* and *MLCK* in intestinal epithelial cells. (**D**,**E**) The protein expression levels of *p*-MLC, occludin, and claudin-1. ** *p* ≤ 0.01, *** *p* ≤ 0.001, ns *p* > 0.05.

The PPI network showed a close association between intestinal immune factors and tight junctions (Figure 5A). Subsequently, the mRNA levels of tight junction-related factors were examined. The mRNA levels of *claudin-1*, *claudin-2*, *claudin-5*, *occludin*, *ZO-1*, and *ZO-3* were decreased in the LPS group compared with the CG. The TL group increased the mRNA levels of these genes compared with the LPS group in tissue and cells (Figure 5B,C). The expression levels of occludin and claudin-1 showed similar trends as the mRNA levels (Figure 5D,E, Figure S3). The MLCK-MLC pathway played a pivotal role in the tight junction of the intestines. In this study, LPS significantly increased the mRNA levels of *MLCK* and *MLC*, which were significantly decreased in the TL group (Figure 5 B,C). The expression levels of *p*-MLC were consistent with the trend in the mRNA levels (Figure 5D,E). These results indicated that TP protected the tight junction in the intestines of common carp.

### 3.6. Radar Plot Analysis of TP on the Mechanism of LPS-Induced Intestinal Injury

**Figure 6:**
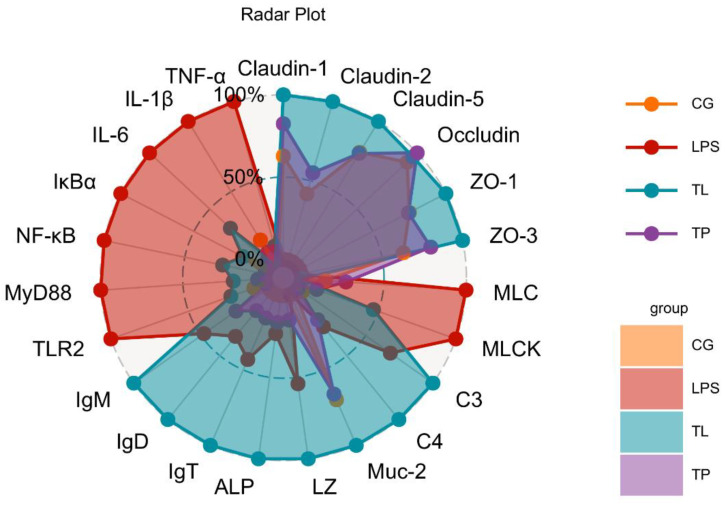
Radar plot of TP on the multiple biomarkers of intestinal damage in common carp. The data of the intestinal immune barrier, inflammation, and tight junction are mapped to coordinate axes, which start from the same center point and end at the periphery of the circle. Orange represents the CG; purple represents the TP group; red represents the LPS group; and blue represents the TL group.

A radar plot was used to visually integrate biomarkers that were detected in this study. Biomarkers accounted for a large proportion of inflammation and tight junction disruption-related pathways in the LPS group. However, biomarkers were mainly concentrated in immune factors and tight junction proteins in the TL group (Figure 6). These results showed that TP activated intestinal immunity and protected intestinal tight junctions to alleviate intestinal injury.

## 4. Discussion

Tea polyphenol, absorbed through the intestine, has great potential in aquaculture. TP can regulate defense mechanisms to indirectly maintain host health [25]. Studies have demonstrated the immunomodulatory effects of tea on fish, such as *Oncorhynchus mykiss*, *Labeo rohita*, and *Epinephelus bruneus* [26,27,28]. LPS plays an important role in pathological damage, inflammation, and immune activation in fish [29]. Therefore, this study established an LPS-induced intestinal injury model to explore the protective effect of TP on the intestines of common carp. H&E results showed that LPS caused severe histopathological damage in the intestine, manifested as shortening and fragmentation of intestinal villi and reduction in muscular layer thickness. TP significantly relieved the intestinal injury, showing intact intestinal villi. Mucosal fold height and muscular layer thickness were significantly higher than those of the LPS group. These results indicated that TP could alleviate LPS-induced intestinal tissue injury.

The immune function of the intestines occupies a key position in the immune defense of teleost. The immune system can directly or indirectly affect muc-2 expression [30]. In this study, LPS induced a decrease in intestinal muc-2 gene expression, while TP could promote the expression of this gene. TP can increase LZ and ALP levels and play an immunostimulatory role in hybrid groupers [15]. Studies have pointed out that dietary Camellia sinensis increases the mRNA level of *LZ* to enhance the fish’s immune system [26]. In this study, TP treatment significantly increased the expression levels of *LZ* and *ALP*, which is consistent with the above studies to a certain extent. Complement is an important component in fighting microbial organisms. C3, the central mediator of the complement system, plays an immune surveillance role [31]. This study found that the addition of TP significantly increased the expression levels of C3 and C4, suggesting that TP stimulates the body to resist LPS challenge. Previous studies have shown that dietary *Jasonia glutinosa* increases the levels of IgM and complement activity in body fluids to stimulate the immune activity of gilthead seabream [32]. In this study, the TL group significantly increased the expression level of IgM, IgD, and IgT, suggesting that TP activated intestinal mucosal immunity. This study reported that TP can improve the immune capacity by regulating the innate and adaptive immune responses in the intestines of common carp.

Inflammatory response and immune regulation are two closely related processes that work together to maintain the stability of the body’s internal environment. Cytokines are playing an important role in regulating intestinal barrier function and intercellular interaction in the intestinal immune system [33]. This study found that TP inhibited the expression levels of inflammatory factors TNF-α, IL-6, and IL-1β, which were increased by LPS. TLRs are major components in the regulation of innate and adaptive immunity [34]. Studies have shown that dietary citrus flavonoids improve immune function by regulating TLR2/NF-κB signaling [35]. Since we found that TP could increase the level of intestinal immunity and inhibit the secretion of inflammatory factors, we hypothesized that TP might exert an immunomodulatory effect by binding to TLR2. Molecular docking showed that the binding energy of TP and TLR2 was −7.9 kcal/mol, indicating that they had strong binding activity. Moreover, the mRNA and protein levels of TLR2 were inhibited by TP in this study. Some studies have found that LPS stimulates NF-κB activation through the TLR2/MyD88 pathway [36]. In this study, LPS also activated the TLR2/MyD88/NF-κB pathway to cause an intestinal inflammatory response. However, TP can significantly inhibit the expression of related proteins, thereby exerting a protective effect. Previous studies have shown that epigallocatechin-3-gallate, the active component of TP, down-regulates NF-κB and TLR signaling to inhibit the production of inflammatory cytokines [37]. The results suggested that TP alleviates LPS-induced intestinal inflammation injury by inhibiting the TLR2/NF-κB pathway. Additionally, the PPI network showed that intestinal immune-related factors were closely associated with tight junctions. NF-κB affects the tight junction barrier by regulating MLCK/*p*-MLC [38]. Studies have shown that *Scytosiphon lomentaria* fucoidan inhibits the TLR4/NF-κB/MLCK pathway to improve tight junction protein levels, protecting tight junction integrity in dietary fiber-deficient mice [39]. In this study, TP inhibited LPS-induced the expression of MLCK and *p*-MLC. Studies have pointed out that luteolin alleviates ethanol-induced tight junction destruction by inhibiting NF-κB nuclear translocation, activating MLCK, and promoting MLC phosphorylation [40]. TP can inhibit TLR4/MLCK signal transduction and protect the pulmonary tracheal epithelial tight junction [41]. MLCK is a key component in regulating tight junction proteins. The upregulation of MLCK can inhibit the mRNA levels of claudins, ZO-1, and occludin [42]. This study revealed that TP alleviated LPS caused by the downregulation of claudins, occludin, and ZO-1 in intestinal tissue and cells. Similar to the present study, dietary TP could inhibit the expression of MLCK and promote the expression of tight junction factors to protect the intestines in the yellow drum [43]. This study found that TP significantly inhibited LPS-induced activation of the NF-κB/MLCK signal to alleviate the disruption of tight junctions in intestinal tissue and cells.

## 5. Conclusions

TP alleviated LPS-induced intestinal injury, protecting the intestinal integrity of common carp. TP could bind to TLR2 to inhibit the expression of NF-κB, activating the intestinal immune capacity. TP further prevented LPS-induced disruption of tight junctions by inhibiting NF-κB/MLCK signal activation. Our study could be valuable for understanding the protective effects of TP on the fish intestines, which supports the regular addition of TP to the diet of fish.

## Data Availability

The original contributions presented in the study are included in the article/Supplementary Materials, further inquiries can be directed to the corresponding authors.

## Supplementary Materials

The following supporting information can be downloaded at: https://www.mdpi.com/article/10.3390/ani15030387/s1, Figure S1: Effects of tea polyphenol (TP) on the viability of primary intestinal epithelial cells; Figure S2: Intestinal morphology indexes of common carp with different treatment; Figure S3: Western blotting images; Table S1: RT-PCR primer information; Table S2: Antibodies used for Western blotting.
