# Supplementary material for: Tea Polyphenol Protects the Immune Barrier and Inhibits TLR2/NF-κB/MLCK Signal Activation to Prevent Inflammatory Injury in the Intestines of Common Carp (Cyprinus carpio L.)
Source: Animals (Basel). 2025 Jan 30;15(3):387. doi: 10.3390/ani15030387 (PMC11815732; doi:10.3390/ani15030387)
Supplement: Supplementary file 1 [file animals-15-00387-s001.zip › animals-3397587-supplementary.pdf]

Tea Polyphenol Protects the Immune Barrier and Inhibits TLR2/NF-κB/MLCK Signal Activation to Prevent Inflammatory Injury in the Intestines of Common Carp (*Cyprinus Carpio* L.)

Man Qian, Jie Yang, Yao Xue, Jiawei Wu, Ziyi Li, Jilong Luo, Bing Zhao * and Xuejiao Gao *

College of Veterinary Medicine, Northeast Agricultural University, Harbin 150030, China; qm18437916979@163.com (M.Q.); yang11jie2022@126.com (J.Y.); 15561850067@163.com (Y.X.); jiayou0609@163.com (J.W.); s230601010@neau.edu.cn (Z.L.); luojilong2024@163.com (J.L.)

* Correspondence: z12zhaobing@163.com (B.Z.); xuejiaogao@126.com (X.G.)

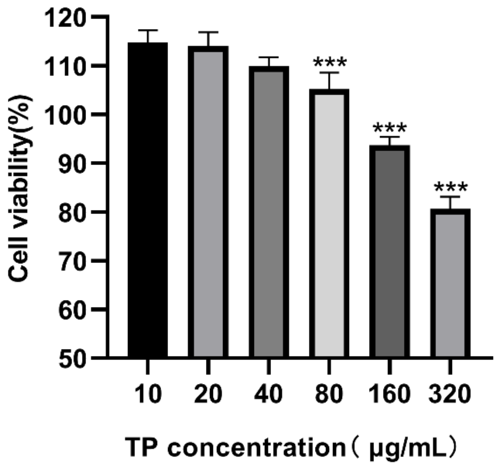

Figure S1. Effects of tea polyphenol (TP) on the viability of primary intestinal epithelial cells. ***$p \leq 0.001$.

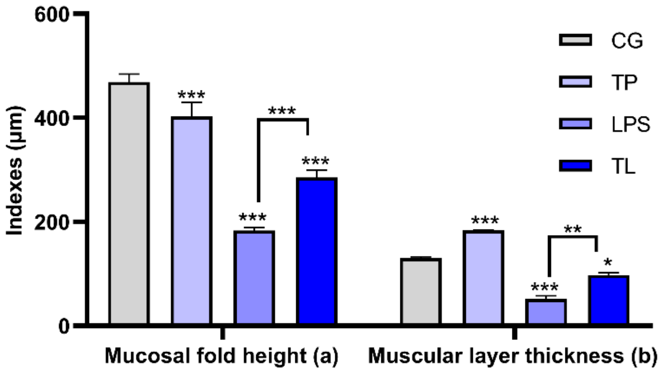

Figure S2. Intestinal morphology indexes of common carp with different treatment. *$p \leqslant 0.05$, **$p \leqslant 0.01$, ***$p \leq 0.001$.

Table S1. RT-PCR primer information.

Gene	Primer sequence (5′-3′)
muc-2	Forward: CAACTAATGCGACTACAACGAATGC
	Reverse: GTTATTCAGTCCAGAGCAGCACAG
ALP	Forward: CCCTCCAAGACCTCAACAAGAATG
	Reverse: TTAGAATGCGTGCTGCTGTGAC
LZ	Forward: GGTGGCGATTGCGGTCTTG
	Reverse: CACATAGTTGCCTAGCGAGAATCC
C3	Forward: CACAGGCGACCATCATAGTATTCC
	Reverse: TCTTCTACCTACCACGGACAGTTC
C4	Forward: CTATGGTGAAGATGTCTCCTGCTATTC
	Reverse: TCTTGTATGTGAGGACTGTGTTGTAAC
IgT	Forward: TCACGCCAACATGGACAATAGAAG
	Reverse:AACACTGAACAACAGATGAACAATGAC
IgD	Forward: TGAACCATCTCCGCCGAAGTC
	Reverse: TCCGTCCATTTGAAAGTAAGAAAGTCC
IgM	Forward: TCTTGGCTTGCTGATGATGAACC
	Reverse: TGATACACGACACAACTGAACACTG
TNF-α	Forward: GATTGCTGCCCTTACCG
	Reverse: CCGCCTTCCTGATTGTT
IL-6	Forward: CGGTGCATTCGATCCTGTTCAAC
	Reverse: GTCTGCGGGTCTCTTCGTGTC
IL-1β	Forward: ACCCGCTGGATTTGTCA
	Reverse: AGGCTCGTCACTTAGTTTGTT
TLR2	Forward: GTGCTCCTGTGAGTTTG
	Reverse: CGACGATTATCCCTTAT
MyD88	Forward: CGCCGAAATGATGGACT
	Reverse: CTGTTGCCTCTGGACGA
NF-κB p65	Forward: AGCAACGACACCACGAA
	Reverse: GGCTCCAGAGGGAACAG
IκBα	Forward: AGAGGCTCGTCATAGTGG
	Reverse: CGATGTGCTCCTGTTTG

Claudin-1	Forward: CCACGAAGGACCGCAAGAAC
	Reverse: ACAGAATATAACCGATGAGCTGAAGAG
Claudin-2	Forward: GTAGCCACTCTCCTGCCATATTG
	Reverse: ACACACTCCATCCATAGACCCTTC
Claudin-5	Forward: TGAAGGCGGAGAGCGTGAAG
	Reverse: AAGTCGGAGATGATGTTGTTGGC
Occludin	Forward: CTGGATATGGAAGTGGATACGGATATG
	Reverse: AATGATGAAGTTTATGGCGGCAATG
ZO-1	Forward: CCTGCCTACACTCAACCACAAC
	Reverse: GCTTCGGCTGGAGGAGGAG
ZO-3	Forward: AGACCACCACCTGAAGATTCCTC
	Reverse: AGAGCGGAGAGTGCCATTAGC
MLC	Forward: CAACCCTACCGCTGATGAAATGG
	Reverse: TCCTCGTATGTTGCCTTGTTTGG
MLCK	Forward: TCACCGTCCTCACAACCTATCAG
	Reverse: ATGCCATGCGACTCTTTCTTCAC
GAPDH	Forward: TACTGGTAGGCCTTCCGTGTCC
	Reverse: CAATTCCGACGCCGACTACCTG

Table S2. Antibodies used for Western blotting.

Antibodies name	Source	Dilution times
p-IκB*α*	ABclonal	1:1000
p-p65	ABclonal	1:1000
MyD88	WanLei Bio	1:1000
TLR2	WanLei Bio	1:1000
p-MLC	ABclonal	1:1000
occludin	WanLei Bio	1:1000
claudin-1	WanLei Bio	1:1000
GAPDH	ABclonal	1:1000
goat anti-mouse IgG-	Proteintech	1:10000

HRP		
goat anti-rabbit IgG-HRP	Proteintech	1:10000

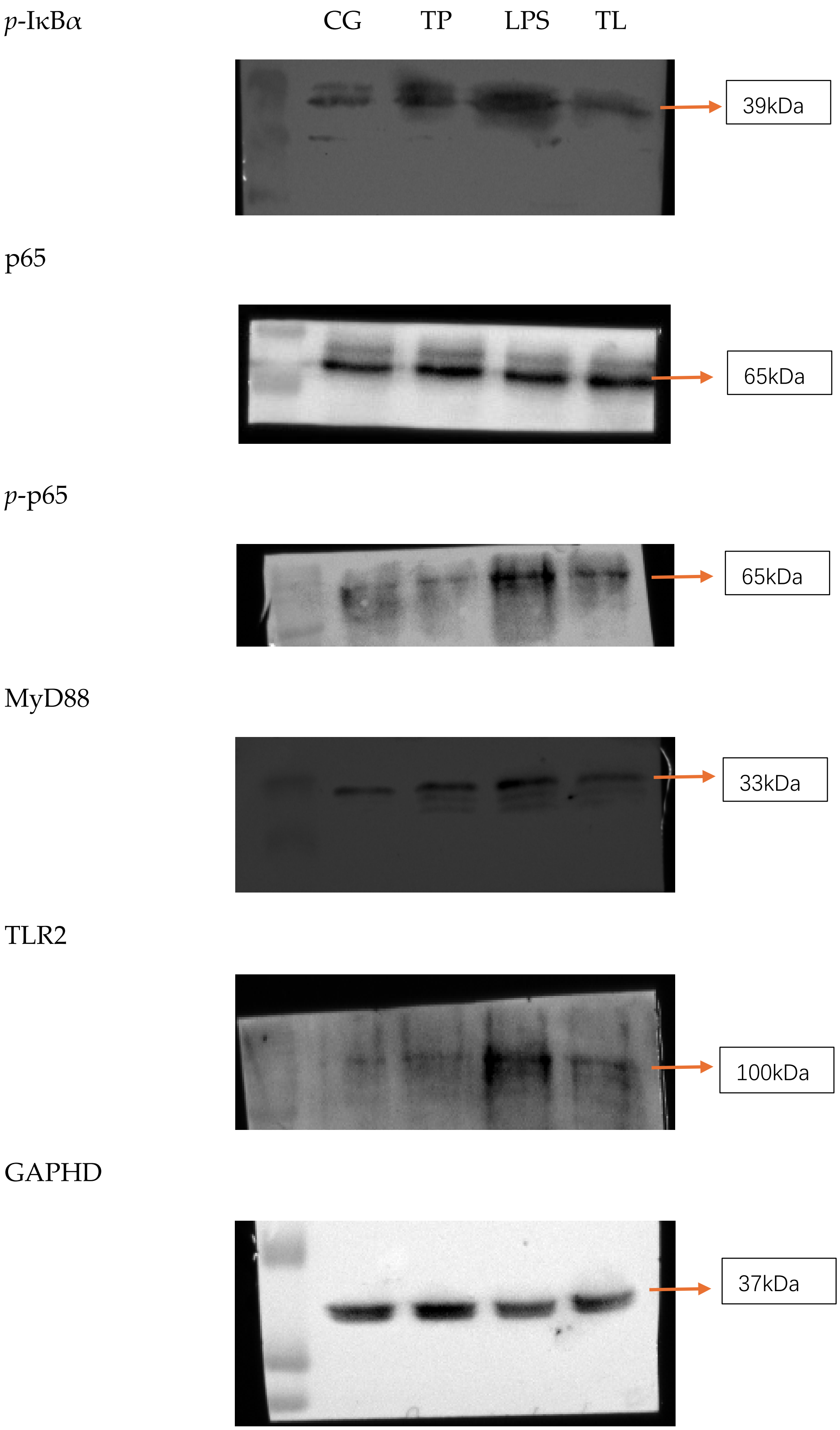

p-IκBα
CG
TP
LPS
TL
39kDa
p65
65kDa
p-p65
65kDa
MyD88
33kDa
TLR2
100kDa
GAPHD
37kDa

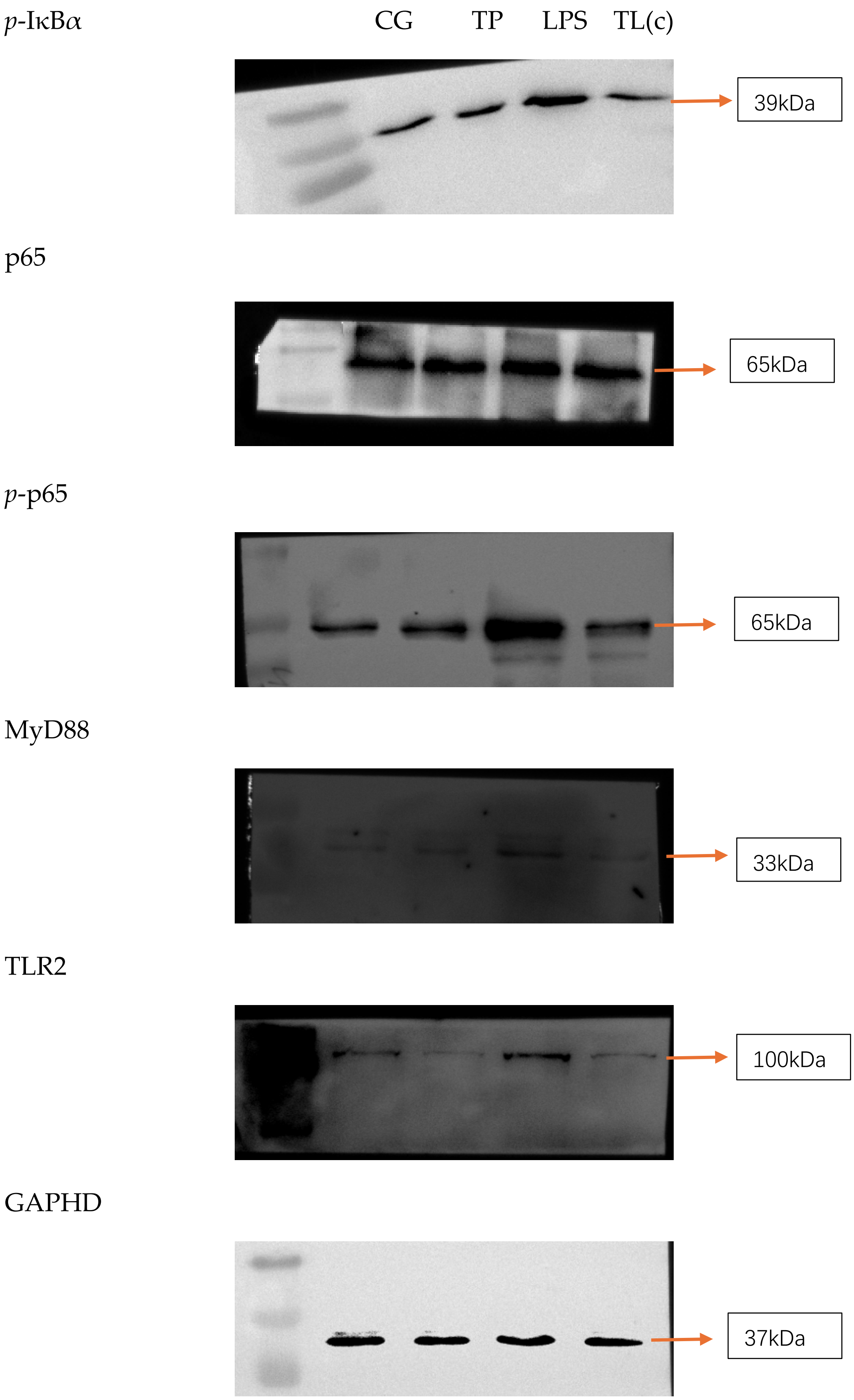

p-IκBα
CG
TP
LPS
TL(c)
39kDa
p65
65kDa
p-p65
65kDa
MyD88
33kDa
TLR2
100kDa
GAPHD
37kDa

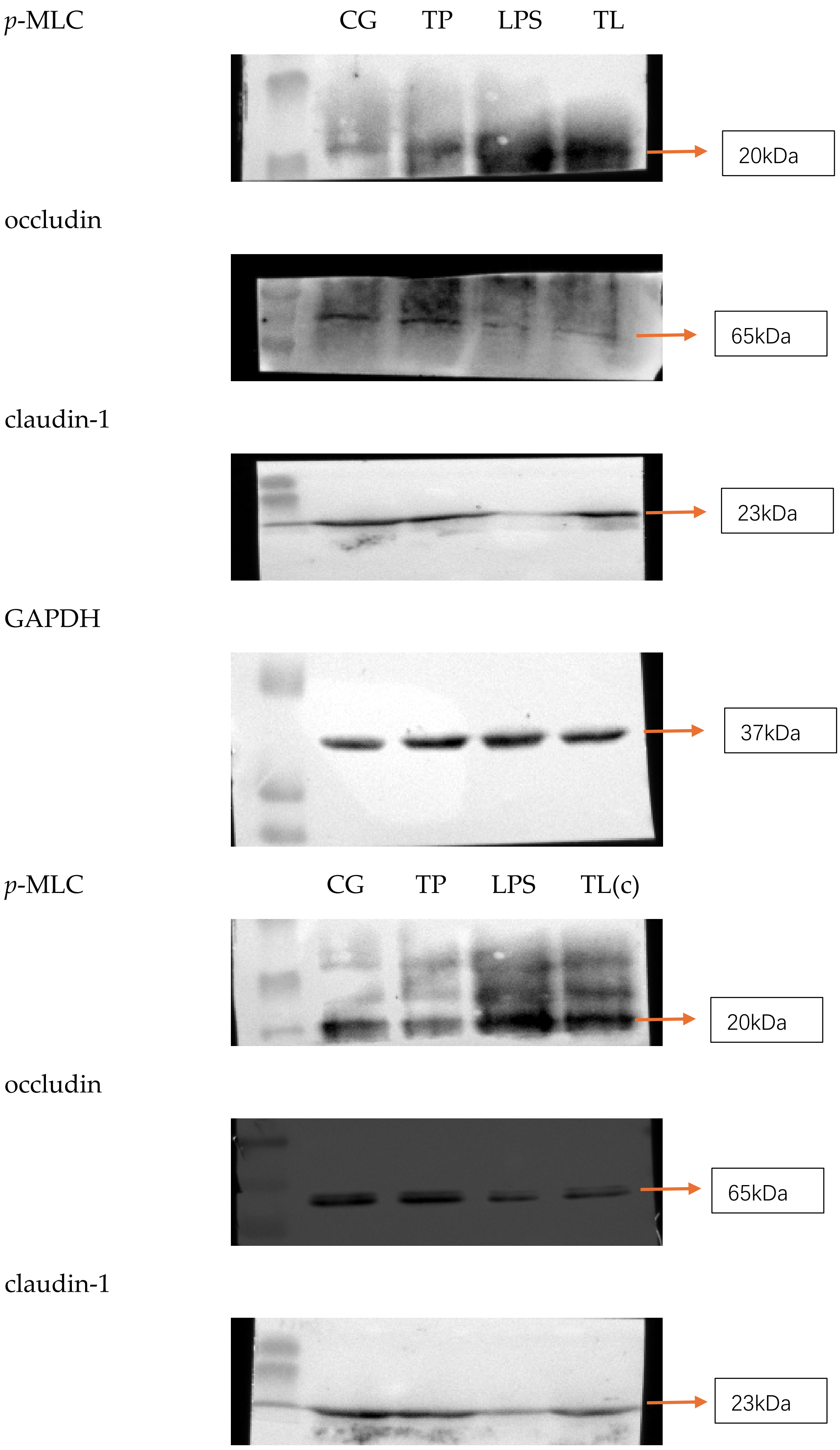
p-MLC
CG
TP
LPS
TL
20kDa
occludin
65kDa
claudin-1
23kDa
GAPDH
37kDa
p-MLC
CG
TP
LPS
TL(c)
20kDa
occludin
65kDa
claudin-1
23kDa

GAPDH

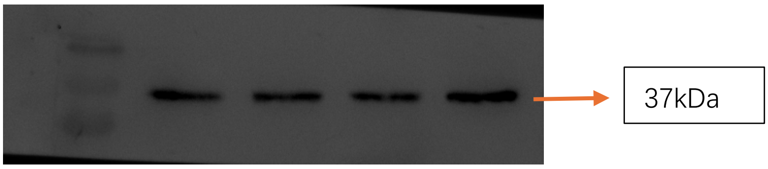

Figure S3. Western blotting images.
